# Knowledge mapping of image-guided tumor ablation and immunity: A bibliometric analysis

**DOI:** 10.3389/fimmu.2023.1073681

**Published:** 2023-02-15

**Authors:** Hui Shen, Lin Wang, Yi Zhang, Guangliang Huang, Baoxian Liu

**Affiliations:** Department of Medical Ultrasonics, the First Affiliated Hospital, Sun Yat-sen University, Guangzhou, China

**Keywords:** tumor ablation, immunity, CiteSpace, VOSviewer, bibliometric

## Abstract

**Background:**

Various ablation techniques have been successfully applied in tumor therapy by locally destroying tumor. In the process of tumor ablation, a large number of tumor cell debris is released, which can be used as a source of tumor antigens and trigger a series of immune responses. With the deepening of the research on the immune microenvironment and immunotherapy, researches exploring tumor ablation and immunity are continuously published. However, no research has systematically analyzed the intellectual landscape and emerging trends for tumor ablation and immunity using scientometric analysis. Therefore, this study aimed to conduct a bibliometric analysis to quantify and identify the status quo and trend of tumor ablation and immunity.

**Methods:**

Data of publications were downloaded from the Web of Science Core Collection database. CiteSpace and VOSviewer were used to conduct bibliometric analysis to evaluate the contribution and co-occurrence relationship of different countries/regions, institutions and authors in the field, and to determine the research hotspots in this field.

**Results:**

By searching in the database, a total of 3531 English articles published between 2012 and 2021 were obtained. We observed rapid growth in the number of publications since 2012. The two most active countries were China and the United States, with more than 1,000 articles. Chinese Academy of Sciences contributed the most publications (n = 153). *Jibing Chen* and *Xianzheng Zhang* might have a keen interest in tumor ablation and immunity, with more publications (n = 14; n = 13). Among the top 10 co-cited authors, *Castano AP* (284 citations) was ranked first, followed by *Agostinis P* (270 citations) and *Chen Qian* (246 citations). According to the co-occurrence and cluster analysis, the results indicated that the focus of research was “photothermal therapy” and “immune checkpoint blockade”.

**Conclusions:**

In the past decade, the neighborhood of tumor ablation domain immunity has been paid more and more attention. Nowadays, the research hotspots in this field are mainly focused on exploring the immunological mechanism in photothermal therapy to improve its efficacy, and the combination of ablation therapy and immune checkpoint inhibitor therapy.

## Introduction

Tumor ablation is a technique that eradicates or substantially destroys the target tumor *in situ* by using energy (ie, thermal and nonthermal) or chemicals (ie, nonenergy) (1). Currently, the most commonly used thermal techniques, are radiofrequency ablation (RFA) and microwave ablation (MWA), which are high-temperature-based modalities, and cryoablation, which is a low-temperature-based modality (2). New technologies such as high-intensity focused ultrasound (HIFU), laser ablation (LA), irreversible electroporation (IRE), plasmonic photothermal therapy, photodynamic therapy and sonodynamic therapy are also applied (3–5). It is undeniable that tumor ablation therapies have a number of advantages, including the minimally invasive nature, the absence of serious systemic side effects, and good healing of the surrounding normal tissues (6). With the further development of medical imaging technology and materials science, ablation technology has achieved great success in the local treatment of tumors (7–10).

Immunity means that the immune system removes alien components or mutated self-components through a series of immune responses of immune cells, to maintain the balance of the immune microenvironment of the body (11). In recent years, with the deepening of immune-related research, the importance of tumor immune microenvironment and cancer immunotherapy has been widely recognized and accepted by researchers (12, 13). Some studies suggest that the tumor immune microenvironment after tumor ablation is carcinogenic to some extent (14–16). In addition, a growing number of studies have demonstrated that tumor ablation can involve tumor antigen release, cross-presentation and the release of damage associated molecular patterns (DAMPs), as well as making the tumor its own cellular vaccine. Tumor tissue destruction by ablation may stimulate antigen-specific cellular immunity engendered by an inflammatory milieu (17). Unfortunately, although tumor ablation can produce a series of tumor-specific immune responses, these immune responses are insufficient to prevent tumor recurrence due to the presence of multiple negative regulators (13). Thus, exploring the neighborhood of tumor ablation and immunity can not only further analyze the changes in the immune microenvironment and the activation/inhibition of immune response during tumor ablation, but also find some potential treatments to improve the efficacy of tumor ablation.

Academic journals have published a big deal of papers exploring tumor ablation and immunity in the past decade. However, no attempts have been made to analyze the data on publications systematically. Bibliometrics is a useful method to probe into the most impactful authors, papers, or countries/regions, construct collaboration networks, and identify research hotspots in particular areas (18, 19). In recent years, numerous bibliometrics studies have been published in journals in different research areas (20, 21). Therefore, we performed a bibliometric analysis to summarize the knowledge base in the field of tumor ablation and immunity and to provide a comprehensive overview of the evidence foundation of tumor ablation and immunity, so that researchers can more easily understand the development of tumor ablation and immunity, and explore the research hotspots in this field.

## Materials and methods

### Data collection

Data between 2012 and 2021 in the field of tumor ablation and immunity were obtained from Web of Science Core Collection (WoSCC) database on August 6, 2022. The database source was limited to Science Citation Index Expanded (SCIE) and publication types to “article”. The main search terms were “tumor ablation”, “immunity”, and “immune response”. The detailed search strategy is presented in the **Supplementary Material 1**. To avoid database update bias, we performed all searches within the same day. Search results were downloaded as “Full Record and Cited References” and “Plain Text”.

### Data analysis

A bibliometric program, VOSviewer (Van Eck and Walt-man, Leiden University, Leiden, The Netherlands) is used to perform the co-occurrence of authors/institutes/countries/journals. In VOSviewer, the size of the node reflects the number of researches or co-occurrence frequencies, the size of the links indicates the co-occurrence frequencies of two nodes, and the same color of node represents the same cluster (22). CiteSpace 5.8.R3 (Chaomei Chen, Drexel University, USA) is a bibliometric software that reveals the trends and dynamics in literature, as well as gives key points in a certain field (23, 24). The CiteSpace parameters were as follows: link retaining factor (LRF = 3), look back years (LBY = 8), e for top N (e = 2.0), time span (2012–2021), years per slice (1), links (strength: cosine, scope: within slices), selection criteria (g-index: k = 25), and minimum duration (MD = 1). We used Python (Python Software Foundation, Wilmington, DE) to draw the world map to show the country distribution of publications. Microsoft Office Excel 2019 (Microsoft, Redmond, Washington, USA) was used to manage the data and analyzed the publication trend. The model (f(x)=p0xn+p1xn−1+p2xn−2+p3xn−3+…+pn) was constructed to predict the number of articles published in 2022.

## Results

### Annual publications and trend

**Figure 1:**
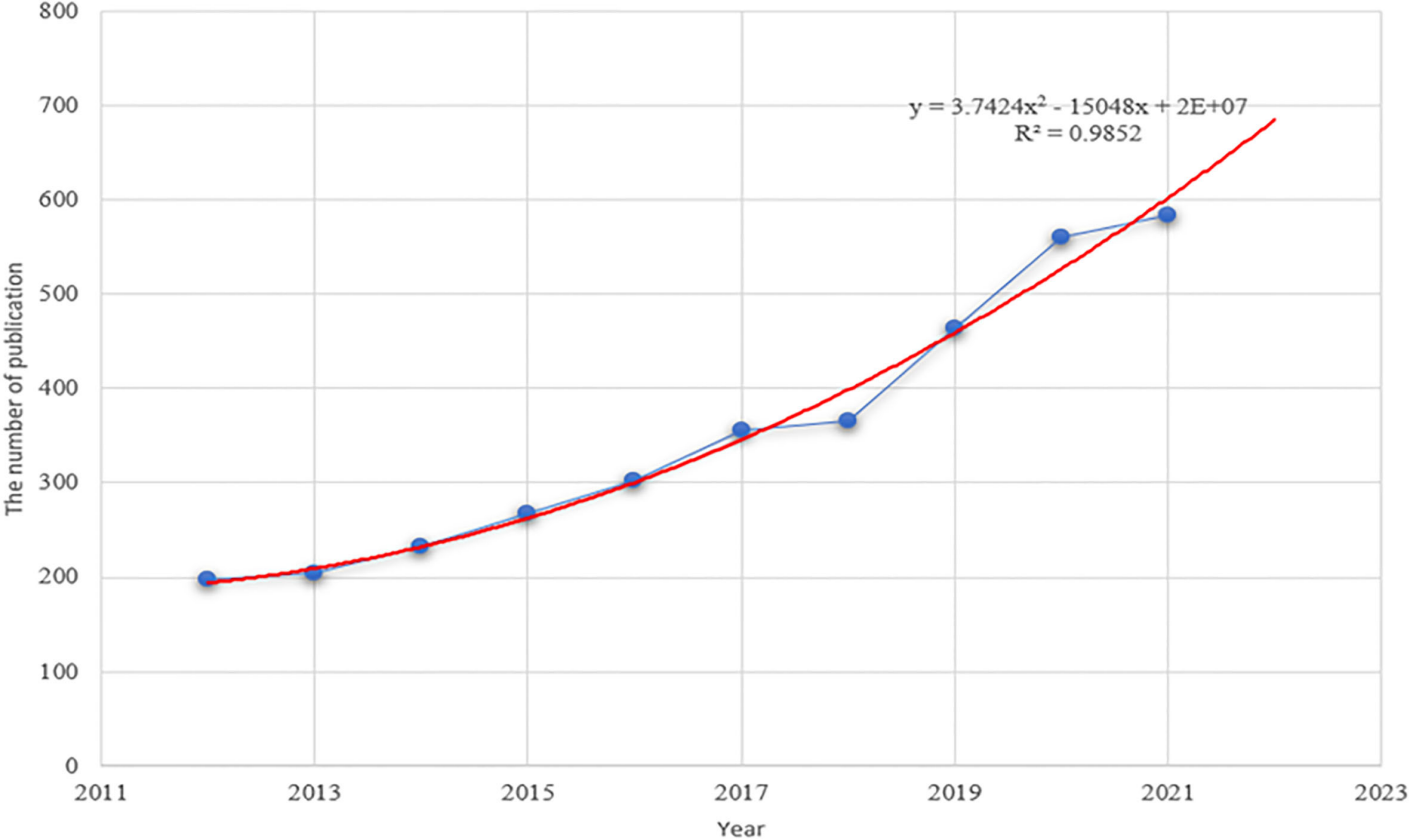
The polynomial curve fitting of publication growth in tumor ablation and immunity.

According to the retrieval criteria, a total of 3,531 papers published between 2012 and 2021 were retrieved from the WOS database. As shown in **Figure 1**, the trend in annual publications consistently kept rising from 197 articles in 2012 to 584 articles in 2021. By constructing the polynomial curve fitting of publication growth in the field of tumor ablation and immunity, we observed a significant correlation between the year and the number of publications (R2 = 0.9852). Through curve fitting, the number of publications about tumor ablation and immunity was estimated to reach 680 in 2022.

### Contribution of countries and institutions

**Figure 2:**
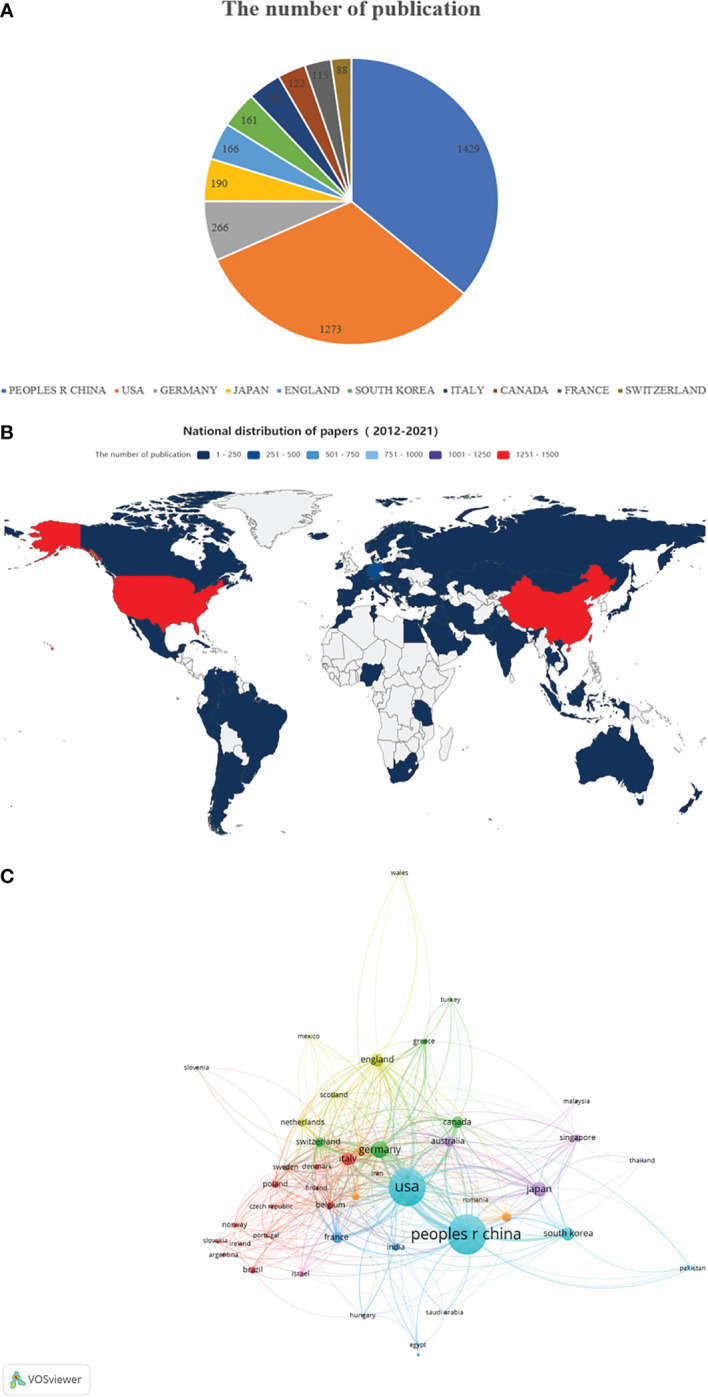
The regional distribution. **(A)** The top 10 countries/regions contributed to the field of tumor ablation and immunity. **(B)** The distribution of countries/regions in terms of publications. **(C)** A network map of countries/regions.

A total of 3,531 articles were from 83 countries/regions and 3121 institutions. **Figure 2A** shows the top 10 most productive countries, while **Figure 2B** shows the world map of publications distribution. China was the foremost productive country, with 1429 articles published, followed by the United States (n=1273) and Germany (n=266). According to the co-authorship network (**Figure 2C**), extensive cooperating relationships were observed among countries/regions. In the network map, it is not difficult to see that China has relatively close cooperation with the United States and South Korea.

**Figure 3:**
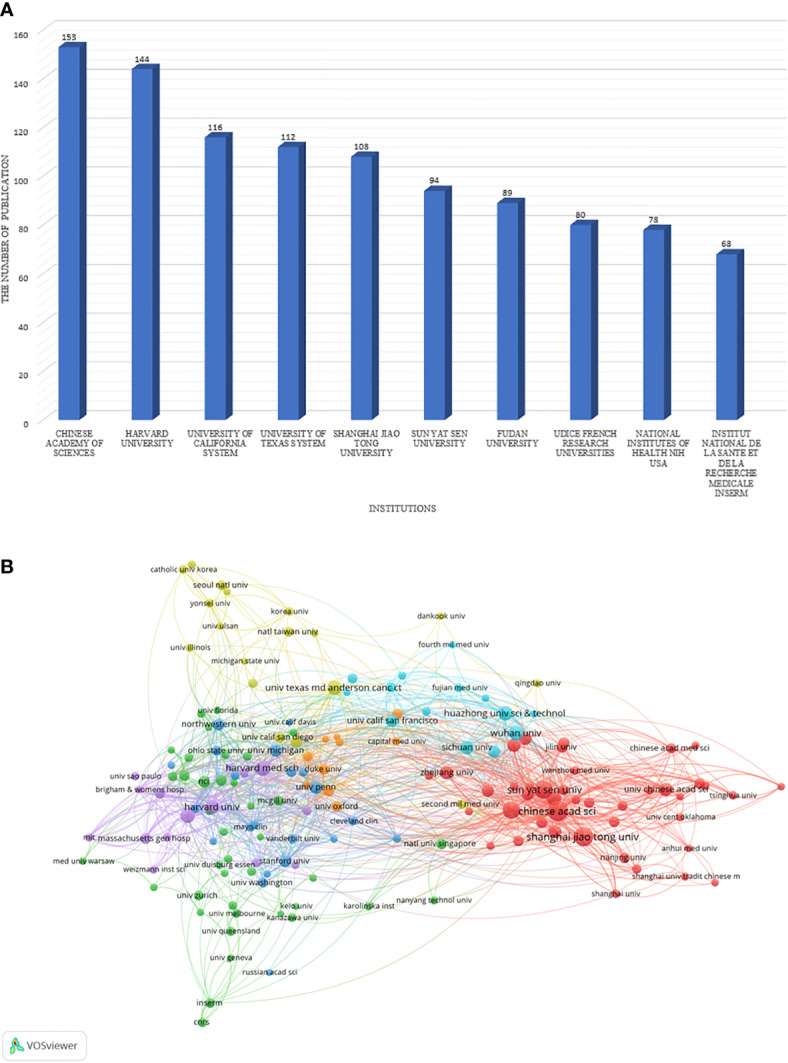
The distribution of institutions. **(A)** The top 10 institutions that published the largest number of articles are listed. **(B)** A network map showing the relations between various institutions.

The top 10 institutions are mainly located in China(n=4), the United States(n=4) and France(n=2) in **Figure 3A**. In addition, the Chinese Academy of Sciences ranks first(n=153), followed by Harvard University(n=144), and the top five institutions all publish over 100 articles. A network map has been created for institutions with more than or equal to 12 (T = 12) publications. As shown in **Figure 3B**, there is rich cooperation between institutions in various countries. For example, the Chinese Academy of Sciences not only has full cooperation with domestic research institutions, but also has close cooperation with the United States, the United Kingdom, Russia and other countries.

### Distribution of authors and co-cited authors

**Figure 4:**
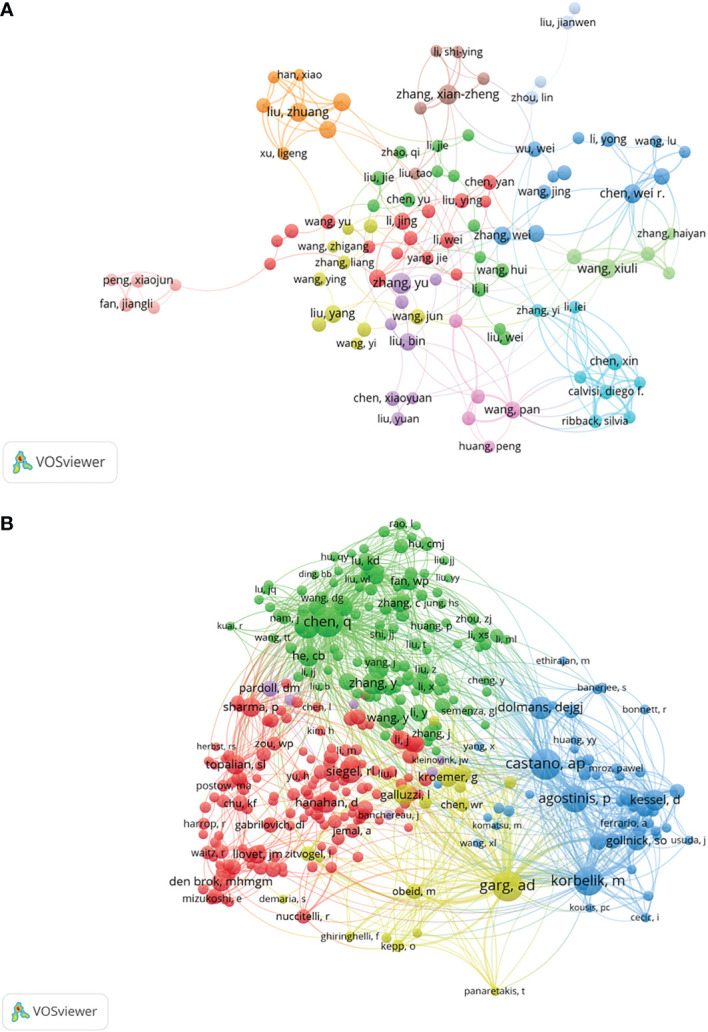
A network map showing authors **(A)** or co-cited authors **(B)** involved in tumor ablation and immunity.

Co-cited authors mean that the authors are cited together (25). More than 20,000 authors and 7,0000 co-cited authors contributed to the investigation on tumor ablation and immunity. **Table 1** shows the top 10 authors and co-cited authors. Of the top 10 contributing authors, *Jibing Chen* (n=14) was ranked first, followed by *Xianzheng Zhang* (n=13) and *Feifan Zhou* (n=13). Among the top 10 co-cited authors, three authors had co-citations over 200. *Castano AP* (284 co-citations) was ranked first, followed by *Agostinis P* (270 co-citations), and *Chen Qian* (246 co-citations). The network map of co-authorship and co-cited relationship were constructed by VOSviewer (**Figure 4**). In **Figure 4**, each node on the network map represents an author, the size of the node means the number of articles, the thickness of the line between the nodes shows collaborative intensity (26). According to **Table 1** and **Figure 4**, *Chen Qian* is not only a productive author and an active co-cited author, but also has close co-cited relationships with other active co-cited authors.

**Table 1:** The top 10 authors and co-cited authors involved in research on tumor ablation and immunity.

Rank	Author	Count	Co-cited Author	Count
1	Jibing Chen	14	Castano AP	284
2	Xianzheng Zhang	13	Agostinis P	270
3	Feifan Zhou	13	Qian Chen	246
4	Zhuang Liu	12	Dolmans DEJGJ	188
5	Yang Liu	12	Siegel RL	179
6	Yu Zhang	12	Garg AD	173
7	Kecheng Xu	12	Zhang Y	161
8	Bin liu	12	Wang C	159
9	Qian Chen	11	Hanahan D	148
10	Wei R Chen	11	Kroemer G	141

### Distribution of journals

In total, 844 academic journals have published research investigating tumor ablation and immunity. In the top 10 journals, 80.00% of the journals published more than 50 articles (**Table 2**). *Cancer Research* (n=82, impact factor (IF) = 13.312) published the most papers, followed by *Plos One* (n=81, IF=3.752), *Photodiagnosis and Photodynamic Therapy* (n=70, IF= 3.577), and *Biomaterials* (n=69, IF = 15.304). Among the top 10 journals, 50.00% had IF higher than 10.000, and *ACS Nano* (n=57, IF = 18.027) is the academic journal with the highest IF.

**Table 2:** The top 10 journals in the field of tumor ablation and immunity from 2012 to 2021.

Rank	Journal	Count	Percent	Country	IF (2021)
1	Cancer Research	82	2.32%	United States	13.312
2	Plos One	81	2.29%	United States	3.752
3	Photodiagnosis and Photodynamic Therapy	70	1.98%	Netherlands	3.577
4	Biomaterials	69	1.95%	Netherlands	15.304
5	Nature Communications	66	1.87%	England	17.694
6	Oncotarget	64	1.81%	United States	NA
7	ACS Nano	57	1.61%	United States	18.027
8	Scientific Reports	53	1.50%	England	4.996
9	Oncogene	43	1.22%	England	8.756
10	Proceedings of the National Academy of Sciences of the United States of America	42	1.19%	United States	12.779

**Figure 5:**
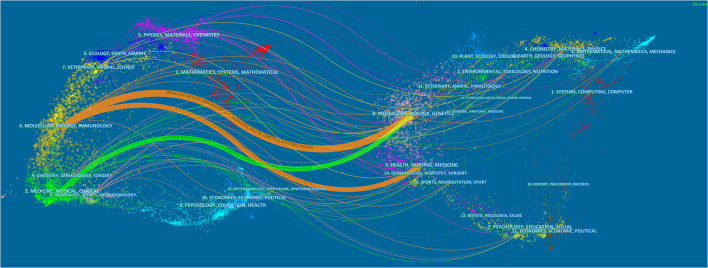
The dual-map overlay of journals related to research on tumor ablation and immunity.

**Figure 5** displayed the dual-map overlay of journals. The left and right sides corresponded to the citing and cited journals, respectively. The label represents the subject covered by the journal and colored curves reflect the citation paths (27). There were three primary citation paths. Two orange citation paths suggested that articles from Molecular/Biology/Genetics journals and Health/Nursing/Medicine journals were frequently cited in articles from the Molecular/Biological/Immunological journals. A green path suggested that articles from the Molecular/Biological/Genetic journals were frequently cited from articles in Medical/Medicine/Clinical journals.

### Reference co-citation analysis

**Figure 6:**
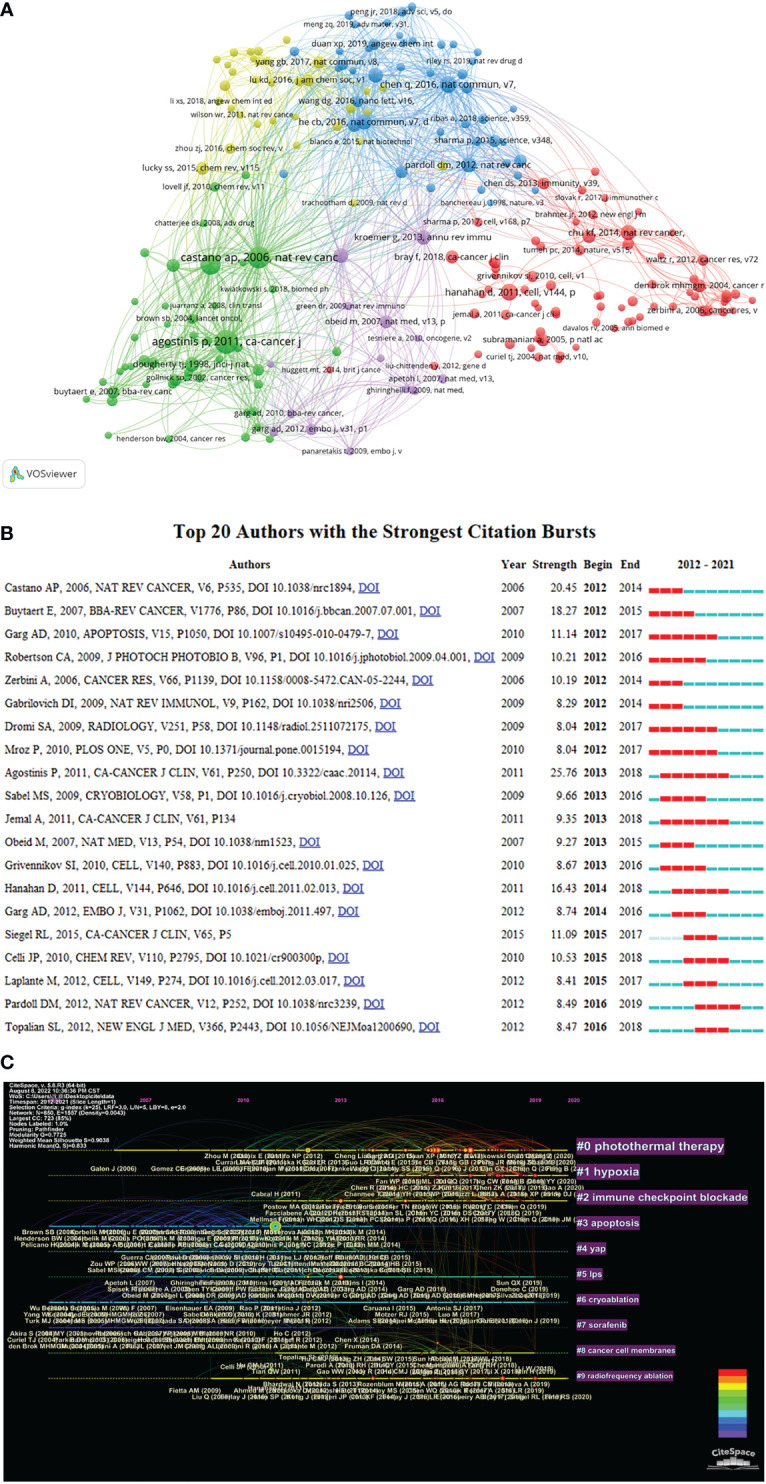
The analysis of co-cited references. **(A)** A network map of co-cited references for research on tumor ablation and immunity. **(B)** Top 20 references with strong citation burstiness. **(C)** The timeline view of co-cited references from publications on tumor ablation and immunity.

Co-citation of reference refers to the relationship between two references cited by the third reference at the same time. **Table 3** presents the top 10 co-cited references related to research exploring tumor ablation and immunity. Among them, the article entitled “Photodynamic therapy of cancer: an update” published in *CA- A Cancer Journal for Clinicians* by *Agostinis P et al.* and the article entitled “Photothermal therapy with immune-adjuvant nanoparticles together with checkpoint blockade for effective cancer immunotherapy” published in *Nature Communications* by *Chen Q et al.* have been co-cited more than 100 times, which indicates that these two articles have a significant impact in the field of tumor ablation and immunity. Besides, VOSviewer is used to construct the network map of co-cited references (**Figure 6A**). In **Figure 6A**, “Agostinis P, 2011, Ca-Cancer J Clin” has a close co-citation relationship with other references with high co-citation times.

**Table 3:** The top 10 co-cited references related to tumor ablation and immunity.

Rank	Co-cited reference	Count	Journals	IF (2021)	Year
1	Agostinis P, 2011, CA-CANCER J CLIN, V61, P250	185	CA-A Cancer Journal for Clinicians	286.130	2011
2	Chen Q, 2016, NAT COMMUN, V7, P0	116	Nature Communications	17.694	2016
3	Chu KF, 2014, NAT REV CANCER, V14, P199	89	Nature Reviews Cancer	69.800	2014
4	He CB, 2016, NAT COMMUN, V7, P0	87	Nature Communications	17.694	2016
5	Xu J, 2017, ACS NANO, V11, P4463	86	ACS Nano	18.027	2017
6	Hanahan D, 2011, CELL, V144, P646	81	Cell	66.850	2011
7	Kroemer G, 2013, ANNU REV IMMUNOL, V31, P51	81	Annual Review of Immunology	32.481	2013
8	Pardoll DM, 2012, NAT REV CANCER, V12, P252	74	Nature Reviews Cancer	69.800	2012
9	Duan XP, 2016, J AM CHEM SOC, V138, P16686	70	Journal of the American Chemical Society	46.802	2016
10	Bray F, 2018, CA-CANCER J CLIN, V68, P394	67	CA-A Cancer Journal for Clinicians	286.130	2018

Reference with citation bursts refers to the reference that is commonly cited by the articles related to the research filed over a period of time. The timeline view in CiteSpace could visually show the changes of research trends over time. In the timeline view, the nodes on the left represent the references with relatively early publication time, and the nodes on the right represent the references with a relatively recent publication time. Additionally, the rightmost label is the cluster label, which reflects the theme of the research field and is ordered from the largest to the smallest number of co-cited references. At the same time, in the timeline, the more nodes there are, the more important the research topic is. CiteSpace is used to search for references with strong citation burst and construct the timeline view of co-cited references (**Figure 6B** and **Figure 6C**). Among the top 20 references with citation bursts (**Figure 6B**), the paper entitled “Photodynamic therapy of cancer: an update” published in *CA: A Cancer Journal for Clinicians* by *Agostinis P et al.*, which is the reference with the strongest citation burst(strength=25.76). As shown in the timeline view (**Figure 6C**), The clusters that are relatively important and have received continued attention include “#0 photothermal therapy”, “#1 hypoxia”, and “#2 immune checkpoint blockade”.

### Analysis of keywords and burst keywords

**Figure 7:**
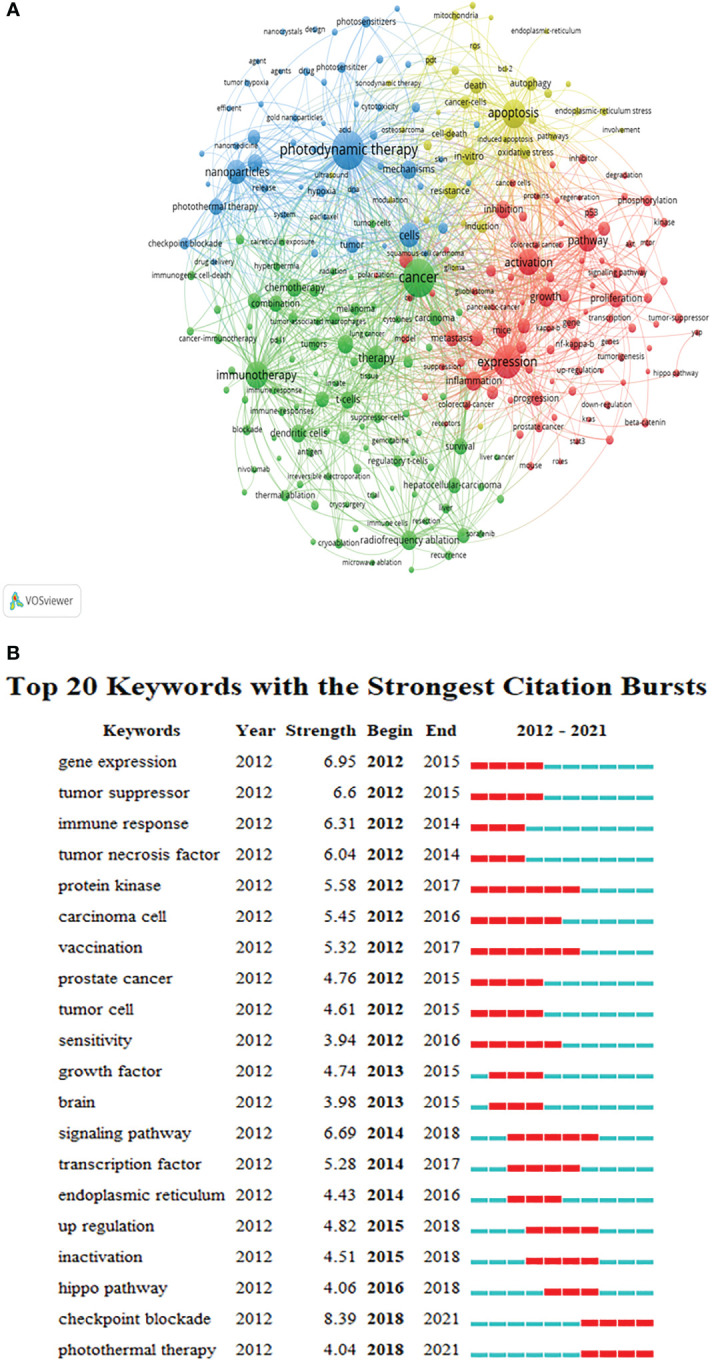
The analysis of keywords. **(A)** A network map of keywords. **(B)** Top 20 keywords with strong citation burstiness.

According to the keyword co-occurrence analysis in VOSviewer, 12,263 keywords were extracted and the network map of keywords was constructed (**Figure 7A**). In **Figure 7A**, the top 5 keywords with the highest rate of occurrence include “photodynamic therapy”, “cancer”, “expression”, “apoptosis” and “immunotherapy”. In addition, the keywords with strong citation bursts were fined through CiteSpace (**Figure 7B**). In recent years, the keywords with strong citation bursts were ‘‘checkpoint blockade’’ (2018–2021, strength 8.39), and “photothermal therapy’’ (2018–2021, strength 4.04).

## Discussion

### General information

According to our research, in the last decade, a total of 3,531 papers have been published in 592 academic journals, with 2,956 authors and 321 institutions from 83 countries/regions based on the WoSCC database. Compared with the number of publications in 2012, the number of publications in 2021 is nearly triple. The growing trend shows that more and more researchers are aware of the importance of the field of ablation and immunity.

Among the top 10 countries in terms of publications, China (n=1429) and USA (n=1273) contributed the most to publications on tumor ablation and immunity, followed by the Germany (n=266) and Japan(n=190). Of the top 10 institutions that published the most research items, China and USA respectively contributed four of the top 10 institutions, followed by the France with two institutions. These findings may suggest that Chinese and American researchers continue to delve into the neighborhood of tumor ablation and immunity. Close collaborations were observed between countries/regions and institutions; however, collaborations between agencies were found to be stronger than those between countries, indicating that international collaborations should be strengthened.

Through the analysis of co-cited authors, we explored the top 10 outstanding researchers in the neighborhood of tumor ablation and immunity. *Castano AP* (284 co-citations) was ranked first, followed by *Agostinis P* (270 co-citations) and *Chen Qian* (246 co-citations). Furthermore, Among the top ten co-cited literature, the first and second-ranked literature were from *Agostinis P* and *Chen Qian*, respectively. As excellent researchers, professor *Castano AP* and *Agostinis P* are devoted to the research of photodynamic therapy. *Castano AP* not only systematically expounded and summarized this therapy, but also explored how photodynamic therapy can kill tumors through immune function (28–30). Meanwhile, *Agostinis P* focused on hypericin photodynamic therapy and explored the potential mechanism of its anti-tumor effect (31, 32). From a comprehensive analysis, professor *Chen Qian* is not only a prolific and highly co-cited author, but also published highly co-cited literature. She is committed to the development of a variety of new nanomaterials to enhance the efficacy of tumor photothermal therapy and immunotherapy to achieve the integration of medicine and engineering (33, 34).

### Research hotspots

By using CiteSpace to capture burst keywords and build the timeline view, we further explored and excavated emerging research hotspots of tumor ablation and immunity. As shown in **Figure 6C** and **Figure 7B**, research hotspots in recent years mainly focus on three aspects. On the one hand, the exploration of a new tumor ablation method-photothermal therapy; on the other hand, the application of immunotherapy in tumor ablation; and finally, the role of hypoxia in the tumor immune microenvironment during ablation.

As we all know, cancer treatment has always been one of the important challenges in clinical work and medical research (35). With the continuous progress of material technology and basic research, in addition to traditional therapy (chemotherapy, radiotherapy and surgery), immunotherapy, targeted therapy and other anti-tumor therapy have been widely used in clinics (36–38). However, patients who receive traditional treatment may have a high risk of treatment failure or side effects after treatment, and targeted therapy is prone to drug toxicity and adverse reactions (39). Photothermal therapy refers to thermal ablation in cancer by generating heat from a photothermal agent. Compared with other tumor ablation methods (such as cryoablation, MWA and RFA), Photothermal therapy has a unique ability. The application of photosensitizers accumulated in tumor tissue provides a certain degree of additional therapeutic selectivity. The controllability of light placement further reduces off-target toxicity in surrounding tissue. The use of interventional techniques with optical fibres and endoscopy further reduces the additional damage caused by invasion (40). Therefore, photothermal therapy as a new way of cancer treatment has gradually attracted more and more attention (41–43). Recently, many studies have shown that hyperthermia produced by photothermal therapy can induce immunogenic cell death (ICD) and release DAMPs or tumor-related antigens, thus increasing the immunogenicity of residual tumor tissues to trigger the immune responses, which shows that photothermal therapy is of great value in remodeling the tumor microenvironment and enhancing tumor immunity for antigenic initiation and metastasis (44). Although photothermal therapy has shown many advantages in the treatment of cancer, its therapeutic effect is still not ideal due to multiple obstacles such as insufficient tumor accumulation, tumor thermal resistance and tumor metastasis (45). Meanwhile, the clinical transformation of photothermal therapy is very slow, and it is still in the preliminary clinical research stage. Consequently, in order to further improve the efficacy of photothermal therapy, it is necessary to further explore the changes of tumor immune microenvironment and the activation of related immune response in photothermal therapy in order to improve its curative effect (46).

In recent years, cancer immunotherapy has not only been paid more attention by the growing number of researchers, but also has been widely used in clinical practice (47, 48). Although a variety of immunotherapy methods are available to treat tumors, including immune checkpoint inhibitors, cytokine, adoptive cell transfer, dendritic cell vaccines and chimeric antigen receptor T cells, immunotherapy based on immune checkpoint inhibitors have become the focus of attention (49, 50). At the same time, tumor ablation has received extensive attention as a promising minimally invasive technique for the treatment of various tumors (2). However, tumor recurrence and distant metastasis after ablation still seem to be the leading cause of death in patients (51, 52). Therefore, how to improve the therapeutic effect and reduce cancer recurrence or metastasis is still a central theme in the field of tumor ablation therapy. Recently, a growing number of studies have shown that ablation therapy could induce or enhance anti-tumor immune response, and ultimately reverse the immunosuppression of tumors to improve the effect of immunotherapy (53). On the one hand, after ablation, a large number of tumor debris and a variety of “danger signals” (such as DAMPs and heat shock proteins) are released, which can be used as antigens and immune stimulators to promote the activation of the immune system (54).At the same time, this activation of the immune system is systemic, which provides the potential for abscopal effect. Many studies have shown that the combination of ablation therapy and immunotherapy amplified the abscopal effect of ablation (55–57). On the other hand, ablation can improve the immune function of lymphocytes, for example, the number of IL-1 β and IL-18 increases after ablation, which can promote the response of type-1 helper T-cell (Th1) and the activation of dendritic cells (DCs) and cytotoxic T lymphocyte (CTLs) (58). Meanwhile, studies have shown that ablation could induce the PD-1/PD-L1 T-cell checkpoint pathway (59). It is not difficult to find that the changes in immune microenvironment after tumor ablation provide the possibility of immunotherapy based on the immune checkpoint inhibitor. Moreover, some emerging ablation technologies are also exploring the possibility of combining with immunotherapy. For example, after photodynamic therapy, various immune cells, including T cells, DCs, neutrophils, NK cells and macrophages are activated, Treg cells are increased and PD-L1 expression is up-regulated (46).At the same time, sonodynamic therapy can trigger systemic immune responses by eliciting ICD and repolarize macrophages from immunosuppressive M2 to antitumor M1 phenotypes (60). Which indicates that the combination of dynamic therapy and immunotherapy is a good strategy. Nowadays, the basic research and clinical research of ablation combined with a variety of immunotherapy including immune checkpoint inhibitors are in full swing, and show a good application prospect (53).

### Strength and limitations

Overall, our study systematically analyzed the field of tumor ablation and immunity by using bibliometric tools, and predicted future research trends. this study not only helps researchers to have a comprehensive understanding of the progress and development of ablation and immunity in the past decade, but also provides ideas for their future research direction.

Inevitably, our study has some limitations. First of all, we only analyzed the data written in English from the WoSCC, excluding data from other important search engines (such as PubMed and Ovid) and other languages. Second, although the WoSCC database is still updating, the study includes the vast majority of articles published in the neighborhood of tumor ablation and immunity between 2012 and 2021, and the new data may not affect the results. Third, we may not include all the tumor ablation methods in the search strategy due to the complexity of ablation techniques, which leads to a certain selection bias in our results. Finally, most of the information was extracted by bibliometrics software, so our results may also be biased. For example, we cannot rule out the possibility that some authors have the same acronym and some keywords have different expressions.

## Conclusion

We used bibliometrics software to conduct a relatively comprehensive and objective analysis of the neighborhood of tumor ablation and immunity, and explored the frontiers of research in this field. In general, exploring the immunological mechanism in photothermal therapy to improve its efficacy, and the combination of ablation therapy and immune checkpoint inhibitor therapy are the main focus of tumor ablation and immunity in the future.

## Data availability statement

The original contributions presented in the study are included in the article/Supplementary Material, further inquiries can be directed to the corresponding author/s. Requests to access the datasets should be directed to shenh25@mail2.sysu.edu.cn.

## Author contributions

HS wrote the paper. GH and BL tracked the paper. LW and YZ collected the data. All authors contributed to the article and approved the submitted version.
