# Supplementary material for: Knowledge mapping of image-guided tumor ablation and immunity: A bibliometric analysis
Source: Front Immunol. 2023 Feb 15;14:1073681. doi: 10.3389/fimmu.2023.1073681 (PMC9975509; doi:10.3389/fimmu.2023.1073681)
Supplement: Supplementary file 1 [file DataSheet_1.docx]

“ablation” OR “high-intensity focused ultrasound ablation” OR “radiofrequency ablation” OR “laser ablation” OR “microwave ablation” OR “cryoablation” OR “photodynamic therapy” OR “irreversible electroporation” OR “ethanol injection” OR “ethanol ablation” OR “HIFU” OR “RFA” OR “MVA” OR “PDT” OR “IRE” OR “PEI” OR “chemical ablation” OR “thermal ablation” OR “percutaneous ablation” OR “radiological ablation” OR “biological ablation”

“immunity” OR “immune” OR “immune processes” OR “immune response” OR “pathway” OR “CD4+ cells” OR " CD8+ cells" OR “t-cells” OR “b-cells” OR “immunology” OR “immune function” OR “immune status” OR “immune cell response” OR “DC cells” OR “NK cells” OR “immune microenvironment” OR “Regulatory T cells” OR “Tregs” OR “Myeloid-derived suppressor cells” OR “MDSCs”

“cancer” OR “carcinoma” OR “carcinoma” OR “tumor” OR “oncology” OR “oncological”
